# High glucose load and endotoxemia among overweight and obese Arab women with and without diabetes: An observational study

**DOI:** 10.1097/MD.0000000000023211

**Published:** 2020-11-13

**Authors:** Dara Al-Disi, Mohammed Ghouse Ahmed Ansari, Shaun Sabico, Kaiser Wani, Syed Danish Hussain, Mona M. Elshafie, Philip McTernan, Nasser M. Al-Daghri

**Affiliations:** a Department of Community Health Sciences, College of Applied Medical Sciences, King Saud University; b Riyadh Biochemistry Department, College of Science, King Saud University, Riyadh, Saudi Arabia; c School of Science and Technology, Department of Biosciences, Nottingham Trent University, Nottingham, NG1 8NS, UK.

**Keywords:** Arab women, endotoxin, high sugar meal, metabolic endotoxemia

## Abstract

Dietary intake influences gut microbiota activity. Nevertheless, there is a lack of evidence available that illustrates the acute effects of high glucose meal on metabolic endotoxemia. The present study assessed the acute impact of high glucose meal on endotoxemia and other clinical parameters in Saudi females with varying degrees of glycemia.

The subjects were 64 consenting pre-menopausal women, grouped into 3: control [n = 14 lean, non-T2DM, BMI = 22.2 ± 2.2 kg/m^2^]; overweight [n = 16, non-T2DM, BMI = 28.5 ± 1.5 kg/m^2^] and T2DM [n = 34, BMI = 35.2 ± 7.7 kg/m^2^]. After an overnight fast, all subjects were given a standardized high-glucose (75 g) meal. Anthropometrics were taken and blood samples were withdrawn at baseline and postprandial (0, 2 and 4-hours), serum glucose, endotoxin and lipid profile were quantified.

At baseline, total cholesterol, LDL-cholesterol, triglycerides and serum glucose levels were significantly higher (*P* values <.01) whereas significantly lower HDL-cholesterol levels (*P* < .01) were observed in T2DM subjects compared to other groups. Baseline endotoxin levels were highest in the overweight group (3.2 ± 1.1 mmol/L) as compared to control (2.0 ± 0.5 mmol/L) and T2DM (2.7 ± 1.2 mmol/L) (*P* = .046). HDL-cholesterol, LDL-cholesterol and triglycerides, significantly decreased in the T2DM group after 2 hours (*P* values <.05), whereas unremarkable changes observed in other groups. Lastly, endotoxin levels significantly increased only in the overweight group (3.2 ± 1.1 vs 4.2 ± 1.4 mmol/L; *P* < .05), 4 hours postprandial.

High glucose meal elevates endotoxemia only among overweight subjects and impairs dysbiosis.

## 1 Introduction

The prevalence of obesity and diabetes across the globe has become the most devastating health impediments that have progressively increased over a decade.^[1–3]^ Numerous studies have endorsed that genetics, lifestyle, dietary habits and environmental factors play a major role in their pathogenesis.^[4–6]^ In recent years, mounting evidence has highlighted that the fluctuations in the gut microbiota (dysbiosis) cause an exponential increase in bacterial endotoxin [lipopolysaccharides (LPS)] levels, which potentiate some inflammatory markers thought to underpin a number of common metabolic diseases.^[7–10]^ Endotoxin translocates from the “leaky” gut lumen to the blood, leading to a condition termed “metabolic endotoxemia,”^[11]^ and elevated circulatory endotoxin levels are associated with an array of chronic non-communicable diseases like type 2 diabetes mellitus (T2DM),^[12]^ obesity,^[13,14]^ atherosclerosis^[15,16]^ and liver disease.^[17]^ Concurrently, several studies have examined the impact of pre-/probiotics on gut microbiota or endotoxins levels and related metabolic diseases.^[7,18,19]^ Furthermore, biomedical industries have shown an intensive commercial interest in the role of the human microbiome and factors alleviating it, by using pre-/pro-biotics.^[20,21]^

It is well known that diet and feeding habits appear to play a predominant role in modulating endotoxin levels over the course of the day.^[22,23]^ Our previous study pointed out that metabolic endotoxemia facilitated by high-fat meal has been exacerbated in subjects with T2DM, accompanied by a parallel rise in cardio-metabolic risk profile compared to their healthy counterparts.^[24]^ This cardiometabolic profile includes lipids such as triglycerides, LDL- and HDL-cholesterol, levels of which are significantly linked to endotoxin-induced sub-chronic inflammation observed in patients with metabolic diseases.^[24]^ To the best of our knowledge, the effect of high–glucose feed on the metabolic endotoxemia, however, remains to be fully understood. Hence, the current study sought to determine the postprandial effects of a high-glucose diet on endotoxemia and other serological parameters in adult Saudi T2DM, overweight females with their healthy counterparts.

## 2 Materials and methods

### 2.1 Participant and study design

In this observational study, 64 Saudi pre-menopausal women were randomly recruited from different primary care centres (PCCs) in Riyadh, Saudi Arabia. A research team member was deployed to each PCC with full knowledge of the protocol to be shared with the assigned PCC physician and nurse. The study has been approved by the Ethics Committee of the College of Medicine King Saud University (No. 10–173), Riyadh, Saudi Arabia, prior to the commencement of the research and all patients gave written consent.

### 2.2 Exclusion criteria

Subjects with chronic conditions such as asthma, hypertension, history of cardiac, kidney or liver disease, in addition, subjects with known long-standing diabetes and/or receiving anti-diabetic medication, those with fasting glucose levels >11 mmol/L, or with fasting triglycerides levels >4 mmol/L were excluded from the study.

### 2.3 Anthropometry and blood collections

Subjects were requested to visit their respective PCCSs after an overnight fast (≥10 hours). Fasting blood samples were collected at baseline to determine subjects eligibility for the study and to assess glucose control as well as lipid profiles. Weight (kg) and height (cm) were recorded using an international standard scale (Digital Pearson Scale, ADAM Equipment Inc., (USA)). Waist circumference was measured at the level of the iliac crest at the end of normal respiration, and hip circumference was measured at the widest circumference around the buttocks using a measuring tape and body mass index (BMI) was calculated as kg/m^2^, as well as waist-to-hip ratios (WHR) were calculated. Blood pressure (mm Hg) was measured twice using a calibrated, mercurial sphygmomanometer, with a 15-minute interval. The mean of the 2 readings was recorded. Based on BMI and DM status, consenting participants were categorized into 3 groups: Control (14 lean, non- T2DM) subjects with body mass index (BMI) 22.1 ± 2.4 kg/m^2^, range 7.8; overweight (n = 16) with 28.5 ± 1.5 kg/m^2^, range 3.6 and subjects with early onset of T2DM (BMI: 35.1 ± 9.0 kg/m^2^; range 35.2, n = 34).

### 2.4 High oral glucose load

Subjects were asked to return the following day, again in a fasted state, to their respective PCC for intervention. Subjects were given a high-glucose meal similar to oral glucose tolerance test (75 g of sugar dissolved in water). They were instructed to fully consume the liquid provided immediately following baseline blood extraction. Blood samples were drawn via peripheral venous can-nula and a flushing line was added to maintain vein patency since blood was collected serially at baseline (0 hours), after 2 hours and 4 hours. Blood samples were collected and transferred to a non-heparinized tube for centrifugation. Collected serum samples were transferred to pre-labelled plain tubes, placed on ice and delivered to the chair for Biomarkers of Chronic Diseases (CBCD) laboratory in King Saud University, Riyadh, KSA, for storage at −20^°^C. All participants were given tokens of appreciation for their participation in the study.

### 2.5 Assessment of biochemical profile

Serum glucose and lipid profile were measured routinely using an autoanalyzer (Konelab, Espoo, Finland). Serum endotoxin was analyzed using a commercially available QCL-1000 LAL End Point.

### 2.6 Data analysis

All statistical analyses were conducted using SPSS version 22.0 (SPSS, Chicago, IL, USA). All continuous variables were presented as mean ± standard deviation and were normalized prior to parametric analyses. For comparison between groups, the univariate general linear model (GLM) was used with Bonferroni post-hoc comparisons. Age and BMI were used as covariates. For comparison between pre- and post-intervention, paired *T*-test was used for normally distributed variables and Wilcoxon Signed Ranks test for endotoxin. Bivariate linear regression analysis was used to determine associations between endotoxin variables of interest. Significance was set at *P* < .05.

## 3 Results

### 3.1 Baseline characteristics

Table 1 shows the baseline characteristics of the 3 groups. The T2DM subjects had significantly higher cholesterol level (5.1 ± 1.2 mmol/L), which was significantly higher from the cholesterol level of control (3.8 ± 0.4 mmol/L) subjects. The concentration of LDL cholesterol was also significantly higher in the T2DM subjects (3.4 ± 1.1 mmol/L) as compared to Overweight (2.6 ± 0.8 mmol/L, *P* value <.01) and control (2.2 ± 0.3 mmol/L, *P* value <.01) subjects. However, the concentration of HDL-cholesterol was significantly lower in T2DM (1.0 ± 0.2 mmol/L, *P* value <.01) and overweight subjects (1.2 ± 0.3 mmol/L, *P* value <.01) as compared to healthy (1.3 ± 0.3 mmol/L) subjects.

**Table 1:** Clinical characteristics of subjects according to the groups.

Parameters	Control	Overweight	T2DM	*P* value
N	14	16	34	
Age (years)	23.2 ± 8.4	33.7 ± 7.6^A^	40.5 ± 5.9^AB^	<.001
BMI (kg/m^2^)	22.1 ± 2.4	28.9 ± 1.3^A^	35.1 ± 9.0^AB^	<.001
Glucose (mmol/L)^∗^	4.4 ± 0.3	4.5 ± 0.5	7.3 ± 1.8^AB^	<.001
Total Cholesterol (mmol/L)	3.8 ± 0.4	4.3 ± 1.0	5.1 ± 1.2^A^	<.001
Triglycerides (mmol/L)^∗^	0.7 ± 0.2	1.1 ± 0.7	1.6 ± 0.6^AB^	<.001
HDL-Cholesterol (mmol/L)	1.3 ± 0.3	1.2 ± 0.3	1.0 ± 0.2^AB^	<.001
LDL-Cholesterol (mmol/L)	2.2 ± 0.3	2.6 ± 0.8	3.4 ± 1.1^AB^	<.001
Endotoxin (mmol/L)^∗^	2.0 ± 0.5	3.2 ± 1.1^A^	2.7 ± 1.2	.046

As expected, subjects with T2DM had significantly higher serum glucose levels (7.3 ± 1.8 mmol/L) than the overweight+ (4.5 ± 0.5 mmol/L, *P* < .01) and control subjects (4.4 ± 0.3 mmol/L, *P* < .001). The subjects with T2DM also had significantly higher serum triglycerides (1.6 ± 0.6 mmol/L). Furthermore, endotoxin levels of the overweight subjects (3.2 ± 1.1) were significantly higher than control subjects (2.0 ± 0.5). However, the endotoxin level of T2DM subjects (2.7 ± 1.2) was not significantly different from either control or overweight subjects.

### 3.2 Effects of high glucose load

Table 2 shows the changes in glucose, lipid and endotoxin profiles of all groups after adjusting for age and metformin. The glucose response to a glucose load was expected to increase in all groups after 2 hours with a concomitant decrease after 4 hours. Among the lipids, there were no significant changes observed in either the control or overweight groups. In the T2DM group, no significant differences in total cholesterol and LDL-cholesterol levels were observed. However, HDL-cholesterol significantly increased in the T2DM group at 4 hours as compared to baseline (*P* < .05) and 2-hours levels (*P* < .05). Furthermore, triglycerides concentration also increased significantly at 4-hours as compared to 2-hours level (*P* < .05). Finally, endotoxin levels significantly increased in the overweight group at 2-hours and 4-hours as compared to baseline endotoxin after adjusting for age and use of metformin (*P* < .05). No significant changes in endotoxin levels were observed in both the control and T2DM group.

**Table 2:** Metabolic changes pre- and post-prandial high-glucose load.

Group	0 – Hour	2 – Hour	4 – Hour
Total Cholesterol (mmol/L)			
Control	3.8 ± 0.4	3.9 ± 0.6	3.8 ± 0.5
Overweight	4.3 ± 1.0	4.3 ± 1.0	4.4 ± 1.1
T2DM	5.1 ± 1.2	4.9 ± 1.1	5.1 ± 1.2
Glucose (mmol/L)^∗^			
Control	4.4 ± 0.3	6.0 ± 1.4	4.7 ± 1.5
Overweight	4.5 ± 0.5	5.8 ± 2.1	4.6 ± 2.9
T2DM	7.3 ± 1.8	13.4 ± 4.5^A^	7.6 ± 4.3^B^
High-density lipoprotein cholesterol (mmol/L)			
Control	1.3 ± 0.3	1.3 ± 0.3	1.3 ± 0.2
Overweight	1.2 ± 0.3	1.2 ± 0.3	1.2 ± 0.3
T2DM	0.97 ± 0.18	0.95 ± 0.18	1.01 ± 0.21^AB^
Low-density lipoprotein cholesterol (mmol/L)			
Control	2.2 ± 0.3	2.2 ± 0.3	2.2 ± 0.3
Overweight	2.6 ± 0.8	2.6 ± 0.8	2.7 ± 0.9
T2DM	3.4 ± 1.1	3.2 ± 0.9	3.3 ± 1.0
Triglycerides (mmol/L)^∗^			
Control	0.7 ± 0.2	0.7 ± 0.4	0.7 ± 0.3
Overweight	1.1 ± 0.7	1.1 ± 0.7	1.1 ± 0.7
T2DM	1.57 ± 0.60	1.49 ± 0.61	1.64 ± 0.71^B^
Endotoxin (EU/ml)^∗^			
Control	2.0 ± 0.5	2.1 ± 0.4	1.6 ± 0.7
Overweight	3.2 ± 1.1	4.0 ± 1.0^A^	4.2 ± 1.4^A^
T2DM	2.7 ± 1.2	2.5 ± 1.3	2.9 ± 1.6

### 3.3 Associations of endotoxins over measured variables over time

**Figure 1:**
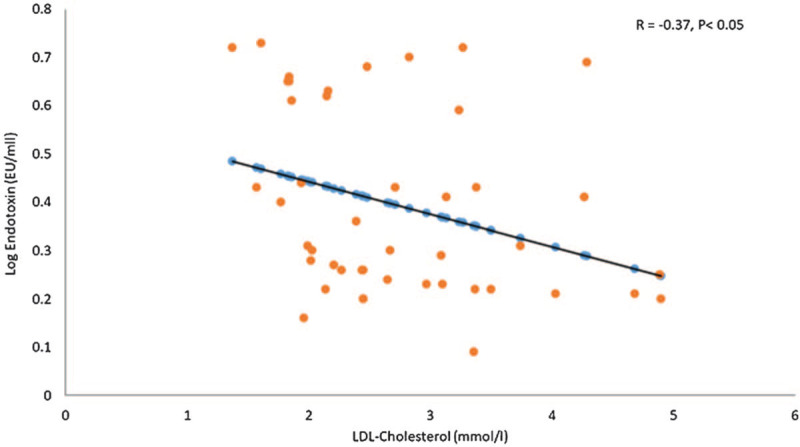
Correlation between log LDL-cholesterol (mmol/L) and Log Endotoxin (EU/ml) at 2-hours in overall patients.

Table 3 shows the bivariate associations of endotoxin to glucose and lipids over time. Endotoxin was negatively associated with LDL-cholesterol only in the overall group and this was observed at 2-hour (R = −0.37; *P* < .05) (Fig. 1). The rest of the associations were non-significant.

**Table 3:** Bivariate associations between lipids, glucose and endotoxin.

Time (Hour)	Total Cholestrol (mmol/L)	Glucose (mmol/L)^∗^	HDL-Cholestrol (mmol/L)	LDL-Cholestrol (mmol/L)	Triglycerides (mmol/L)^∗^
Overall (N = 64)					
0 – Hour	0.04	0.17	0.26	−0.12	0.12
2 – Hour	−0.24	−0.25	0.21	−0.37^†^	0.10
4 – Hour	−0.09	0.21	−0.11	−0.16	0.27
Control (N = 14)					
0 – Hour	0.43	0.49	0.42	−0.27	0.40
2 – Hour	0.20	0.37	0.47	−0.43	0.37
4 – Hour	−0.33	0.07	−0.29	−0.36	0.19
Overweight (N = 16)					
0 – Hour	0.13	0.46	0.48	0.06	−0.46
2 – Hour	−0.21	0.43	0.25	−0.61	0.43
4 – Hour	−0.23	0.37	−0.05	−0.05	0.28
T2DM (N = 34)					
0 – Hour	0.04	0.36	0.15	−0.04	0.23
2 – Hour	−0.19	−0.03	0.11	−0.28	0.17
4 – Hour	−0.16	0.37	−0.05	−0.27	0.36

## 4 Discussion

The main finding in the present study is that circulating levels of endotoxin are elevated after a high glucose meal among overweight non-T2DM subjects and these unfavorable changes were not observed among non-overweight, non-T2DM as well as T2DM subjects. The significant increase of endotoxin levels among overweight subjects suggests exacerbation of an already dysfunctional gut among these subjects. Physiologically, endogenous insulin released into the circulation as a response to acute hyperglycemia.^[25]^ During this process and in the presence of a healthy gut such as the case of controls in the present study, no aberrant changes will be observed in endotoxin levels. In the case of overweight subjects, however, the significant increase in endotoxin levels following a high glucose load implicates the presence of insulin-resistance and probably systemic low-grade chronic inflammation within this cohort, making the leaky gut even leakier. Chronic exposure to endotoxin may increase T2DM risk since higher levels of endotoxin, in general, is associated with insulin resistance.^[11]^ However, only a few studies have evaluated the influence of nutrients and/or specific food types on endotoxemia.^[26]^

In our study, endotoxin levels following a high-glucose meal showed a dramatic increase only in the overweight and not in the T2DM group. Among several possibilities, is the lack of any reduction in endotoxin levels in the T2DM group may be due to the differences in the subjects dietary make-up. The main effect, however, might be the medications that this group has been taking which include metformin. Metformin has been shown to drastically improve the gut microbiota composition among T2DM.^[5,27,28]^ This could probably explain why endotoxin at baseline for the T2DM group is much lower than the overweight group and why the response to a glucose load was almost as similar to controls. The significant increase in endotoxin observed in the overweight group can be explained by an already existing insulin-resistant state in the subjects, as evidenced by the modest but significantly higher baseline endotoxin levels compared to other groups.

Finally, in the present study, we observed a significant inverse association between endotoxin and post-prandial LDL-cholesterol. While no significant differences were observed between LDL-cholesterol over time in all groups, most studies indicate LDL-cholesterol levels to be lower postprandial as compared to fasting levels, at least in people with T2DM or with insulin resistance.^[29]^ This could explain why increasing endotoxin levels, particularly in the overweight and T2DM groups, translated to lower LDL-cholesterol levels over-all. The lack of associations between endotoxin and other cardiometabolic parameters in the present study does not supersede our previous findings on the strong relationship between endotoxin and these measures after high fat intake,^[30]^ and the lack of associations may be due to the sample size issues.

The authors acknowledge some limitations and these include the small sample size and other factors not documented in the study such as lifestyle and mode of nutrition, which can significantly modify the gut microbiome. Further and longer prospective investigations using the full components of metabolic syndrome,^[31]^ with a larger number of subjects and inclusion of more inflammatory markers may provide clear insights into how glucose-induced endotoxemia affect the over-all metabolic picture of non-T2DM overweight subjects. Lastly, Arab women are more prone to develop obesity secondary to religious and cultural norms prevalent in the region, making them at higher risk for T2DM and therefore the study focused on this population. Whether the same altered endotoxin levels apply to men of varying glycemic levels need to be investigated separately. The study is nevertheless the first to explore the effects of high glucose load in endotoxin levels among Arab ethnic women with varying levels of glycemia. This has clinical implications, especially in the region where the concept of gut microbiota and endotoxin in particular, and how it affects obesity-related diseases, remain to be explored.

## 5 Conclusions

In conclusion, an acute high glucose load promotes endotoxemia among overweight subjects and exacerbates dysbiosis. This effect is not observed among T2DM patients on metformin as well as non-obese, non-T2DM subjects.

## Acknowledgments

The authors thank the volunteers and research team from the different primary care centers for the recruitment of subjects.

## Author contributions

“Conceptualization, D.A and P.T.; methodology, D.A. and M.M.E; formal analysis, S.D.H. investigation, D.A., M.G.A.A., M.M.E; S.S.; K.W.; resources, N.M.A.; data curation, K.W.; writing—original draft preparation, M.G.A.A.; writing—review and editing, S.S., P.T; supervision, N.M.A.; project administration, D.A; funding acquisition, N.M.A. All authors have read and agreed to the published version of the manuscript.

**Conceptualization:** Dara Al-Disi, Philip McTernan.

**Formal analysis:** Syed Danish Hussain.

**Funding acquisition:** Nasser Al-Daghri.

**Investigation:** Dara Al-Disi, Shaun Sabico, Kaiser Wani, Mona M. Elshafie.

**Methodology:** Mohammed Ghouse Ahmed Ansari, Shaun Sabico, Kaiser Wani, Mona M. Elshafie.

**Supervision:** Philip McTernan, Nasser Al-Daghri.

**Writing – original draft:** Mohammed Ghouse Ahmed Ansari.

**Writing – review & editing:** Dara Al-Disi, Shaun Sabico, Kaiser Wani, Syed Danish Hussain, Mona M. Elshafie, Philip McTernan, Nasser Al-Daghri.
